# Functional dissection of the *Drosophila *Kallmann's syndrome protein DmKal-1

**DOI:** 10.1186/1471-2156-7-47

**Published:** 2006-10-11

**Authors:** Davide Andrenacci, Maria R Grimaldi, Vittorio Panetta, Elena Riano, Elena I Rugarli, Franco Graziani

**Affiliations:** 1 Institute of Genetics and Biophysics, A. Buzzati Traverso, CNR, 80131 Napoli, Italy; 2 Telethon Institute of Genetics and Medicine, 80131 Napoli, Italy; 3 Division of Biochemistry and Genetics, Istituto Nazionale Neurologico "C. Besta", 20126 Milano, Italy; 4 Present address: Dipartimento di Biologia e Patologia Cellulare e Molecolare "L. Califano", Università Federico II, 80131 Napoli, Italy

## Abstract

**Background:**

Anosmin-1, the protein implicated in the X-linked Kallmann's syndrome, plays a role in axon outgrowth and branching but also in epithelial morphogenesis. The molecular mechanism of its action is, however, widely unknown. Anosmin-1 is an extracellular protein which contains a cysteine-rich region, a whey acidic protein (WAP) domain homologous to some serine protease inhibitors, and four fibronectin-like type III (FnIII) repeats. *Drosophila melanogaster *Kal-1 (DmKal-1) has the same protein structure with minor differences, the most important of which is the presence of only two FnIII repeats and a C-terminal region showing a low similarity with the third and the fourth human FnIII repeats. We present a structure-function analysis of the different DmKal-1 domains, including a predicted heparan-sulfate binding site.

**Results:**

This study was performed overexpressing wild type DmKal-1 and a series of deletion and point mutation proteins in two different tissues: the cephalopharyngeal skeleton of the embryo and the wing disc. The overexpression of DmKal-1 in the cephalopharyngeal skeleton induced dosage-sensitive structural defects, and we used these phenotypes to perform a structure-function dissection of the protein domains. The reproduction of two deletions found in Kallmann's Syndrome patients determined a complete loss of function, whereas point mutations induced only minor alterations in the activity of the protein. Overexpression of the mutant proteins in the wing disc reveals that the functional relevance of the different DmKal-1 domains is dependent on the extracellular context.

**Conclusion:**

We suggest that the role played by the various protein domains differs in different extracellular contexts. This might explain why the same mutation analyzed in different tissues or in different cell culture lines often gives opposite phenotypes. These analyses also suggest that the FnIII repeats have a main and specific role, while the WAP domain might have only a modulator role, strictly connected to that of the fibronectins.

## Background

Kallmann's syndrome (KS) is a heritable disorder characterized by the association of anosmia or hyposmia, i.e. the lack or reduction of the sense of smell, and hypogonadotropic hypogonadism [1]. These anomalies probably arise from impaired targeting and migration of the olfactory axons and of the neurons secreting the gonadotropin-releasing hormone (GnRH), both originating in the olfactory placode [2,3], or from alterations in the initial steps of olfactory bulb differentiation [4]. KS patients have aplasia or hypoplasia of olfactory bulbs and tracts, and with less frequency display other symptoms, such as mirror movements, unilateral renal aplasia and cleft lip/palate [5-7]. Notably, some of these symptoms are attributable to defects in morphogenesis.

Up to now, only two of the genes involved in KS have been identified: *KAL-1 *and *KAL-2*. *KAL-1 *is responsible for the X-linked form of the disease and encodes an extracellular matrix protein. This protein (anosmin-1) has a peculiar domain composition, with a cysteine-rich (CR) region at the N-terminus, followed by a whey acidic protein (WAP) domain and four fibronectin-like type III (FnIII) repeats [8,9]. *KAL-2 *is the gene responsible for an autosomal dominant form of KS and encodes the fibroblast growth factor type one receptor (FGFR1) [10]. Together, mutations in these two genes account for approximately 30% of KS cases.

Functional studies on the role of *KAL-1 *have been hampered by the failure to identify a mouse ortholog, although *KAL-1 *homologs have been found in many different species, including invertebrates [11-13]. Experimental evidences in different systems indicate that anosmin-1, secreted by neurons in the olfactory bulb, is a key factor controlling successful innervation and organization of the olfactory bulbs [14-18] and migration of GnRH neurons [19]. Studies in *C. elegans *demonstrated a role of the *kal-1 *gene in both epithelial morphogenesis and neurite outgrowth and branching. Notably, these processes were affected in *kal-1 *loss of function mutant but also when *kal-1 *was overexpressed [11,12]. Using the overexpression approach, it has also been demonstrated that a single amino acid substitution in the first FnIII repeat, which reproduces a mutation found in a KS patient [20], abolished the axon branching activity but had no effects on axon targeting activity of the protein. Another mutation, which disrupted two of the four disulfide bonds of the WAP domain, did not impair the overall branching propensity of *kal-1 *but it abolished the potential to misroute axons [12,21]. On the basis of these results, it was suggested that the branching activity and the outgrowth activity of CeKal-1 are genetically separable.

In *Drosophila *only one *kal-1 *ortholog was found, which encodes a protein smaller than vertebrate and worm orthologs [12,13]. *Drosophila melanogaster *Kal-1 (DmKal-1) has only two FnIII repeats, while the C-terminal region displays low similarity with the third (32%) and the fourth (36%) human FnIII repeats. During *Drosophila *embryonic development, *kal-1 *is expressed in a complex and dynamic pattern in cells involved in morphogenetic processes or associated with sensory organs and is also expressed in male-specific somatic gonadal precursors (msSGPs) [22]. *kal-1 *is strongly expressed in some ectodermal cells of the mandibular segment, which are probably involved in the formation of the head skeleton of the embryo. In fact, *kal-1 *expression is reduced or abolished in *Deformed *(*Dfd*) and *cap-n-collar *(*cnc*) homeotic mutants, which lack some components of the anterior cephalopharyngeal skeleton [13].

In this article, we present a systematic structure-function study of DmKal-1 using an overexpression approach. We show that DmKal-1 can cause strong alterations in the cephalopharyngeal skeleton of the larva when it is expressed in cells that surround the head skeleton, suggesting a role of *kal-1 *in the morphogenesis of this organ. We did not characterize in further detail the possible function of *kal-1 *in the formation of the head skeleton, but we utilized this phenotype to perform an *in vivo *molecular dissection of the contribution played by the different domains of the protein, expressing a series of mutated forms of DmKal-1. In this analysis we looked for qualitative differences in the phenotypes induced by the expression of the different mutant forms. We have also expressed the wild type and the mutated DmKal-1 proteins in another tissue, to test whether the functional relevance of each single domain depends on the extracellular context. We selected the adult wing, because it is very easy to detect even small defects caused by alterations in the morphogenetic processes leading to the final structure of this organ. We have found that the expression of wild type DmKal-1 causes several alterations in the wing development, while the defects induced by the mutants suggest that the activity of the protein is dependent on the extracellular context. The binding activity of mutations in the first FnIII repeat were also analyzed by *in vitro *transfection experiments in COS-7 cells.

## Results

### *kal-1 *overexpression during the cephalopharyngeal skeleton formation determines alterations of the head skeleton structure

**Figure 1:**
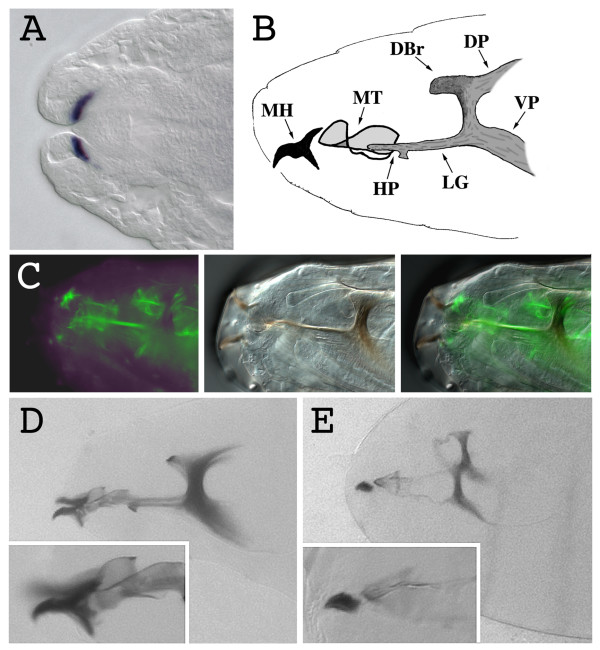
***kal-1 *overexpression in the cells responsible for the formation of the cephalopharyngeal skeleton causes alterations of the sclerotized components of the head**. (A) Stage 16 wild type embryonic head. The *kal-1 *transcript is detected in the region of the gnathal lobes that will give rise to the anterior part of the cephalopharyngeal skeleton. (B) Schematic representation of the cephalopharyngeal skeleton of a L1 *Drosophila *larva. MH mouth hook, MT median tooth, HP H-piece, LG lateralgräten, DBr dorsal bridge, DP dorsal process, VP ventral process. (C) Fluorescence (left), bright-field (center), and merged (right) images of the expression in the cephalopharyngeal skeleton of a stage 17 embryo of the UAS-*GFP *reporter, driven by the *179y*-Gal4 line. (D) Wild type stage 17 embryonic head cuticle. In the inset, a magnification of the mouth hooks. (E) Head cuticle of a stage 17 *179y*-Gal4/+; UAS-*kal-1*/+; UAS-*kal-1*/+ embryo. The head skeleton structure appears less sclerotized; principally the median tooth, the H-piece, and the lateralgräten, but also the dorsal bridge, the dorsal process, and the ventral process appear thinner than in the wild type. The mouth hooks lack the posterior part (inset).

**Figure 2:**
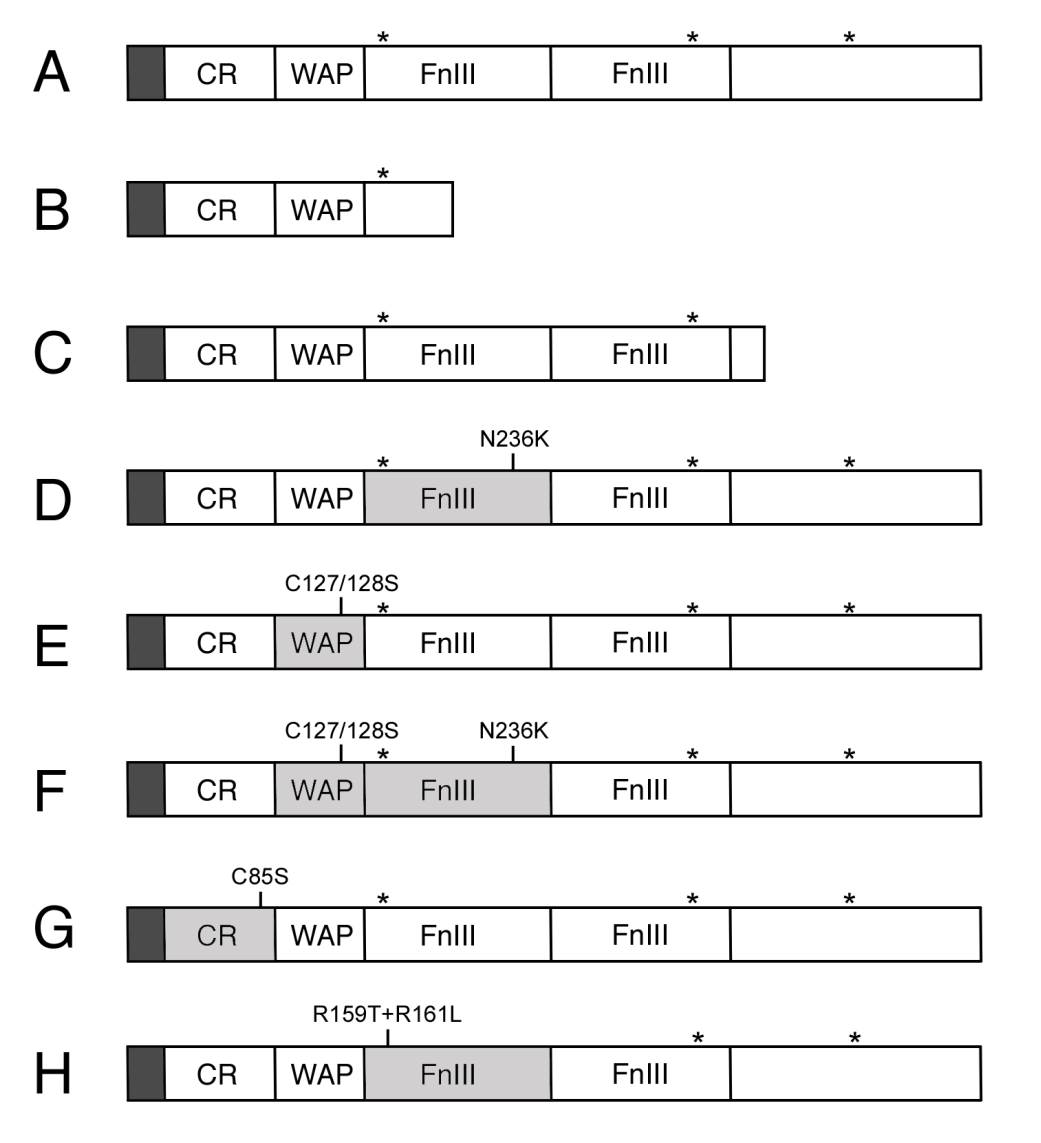
**Schematic structure of Dmkal-1 and of the different mutant proteins analyzed**. (A) Wild type Dmkal-1. (B) Dmkal-1 [W200-STOP]. (C) Dmkal-1 [H384-STOP]. (D) Dmkal-1 [N236K]. (E) Dmkal-1 [C127/128S]. (F) Dmkal-1 [C127/128+N236K]. (G) Dmkal-1 [C85S]. (H) Dmkal-1 [R159T+R161L]. CR, Cysteine-rich domain; WAP, Whey Acidic Protein-like domain; FnIII, Fibronectin-like type III domain. Asterisks indicate the position of heparan-sulfate binding site consensus sequences. In gray the domains affected by mutations.

*kal-1 *is expressed in the second part of embryogenesis in cells involved in morphogenetic processes such as germ band retraction (GBR), dorsal closure (DC) and head involution (HI) [13]. We found a strong expression in a restricted group of ectodermal cells in the mandibular segment during HI (Fig. 1A) [13], suggesting an involvement of *kal-1 *in this morphogenetic process. The mandibular segment gives rise to the anterior part of the cephalopharyngeal skeleton, comprising the mouth hook base and the lateralgräten [23]. To test a possible function of *kal-1 *in the formation of these structures, we overexpressed it in the cells responsible for the formation of the head skeleton. To this purpose, we prepared transgenic lines that express the *kal-1 *cDNA under the control of the UAS promoter (P [UAS-*kal-1*]) (Fig. 2A) [24] and crossed them with the *179y*-GAL4 line [25]. In this transgenic line, the transactivator is expressed during the second part of embryogenesis, showing a ubiquitous distribution from stage 13 to stage 16. In particular, during stages 15 and 16, the GAL4 protein is abundant in the whole ectoderm (data not shown). At stage 17, when the sclerotization of the mouth parts takes place, the expression of *179y*-GAL4 is detectable at the base of the mouth hooks and in the cells surrounding the whole head skeleton (fig. 1B, C). The P [UAS-*kal-1*] line was crossed with *179y*-GAL4 and we found that a consistent amount of eggs failed to hatch (43%, n 226), while the surviving larvae showed a delay of the hatching (data not shown). Using a transgenic line with P [UAS-*kal-1*] insertions on the second and the third chromosome (P [UAS-*kal-1*]^II and III^), we obtained a stronger phenotype with a greater amount of non hatching larvae (72%, n 193), suggesting a dose dependent effect of the expression of *kal-1 *on the hatching process.

Cuticle preparations of embryos from the P [UAS-*kal-1*]^II and III ^line revealed appreciable head skeleton alterations, but no other defects. The mouth hooks of a substantial portion of *kal-1 *expressing larvae lacked the sclerotic material that forms the base of the hook. The dorsal and ventral processes of the hooks were variably reduced, leaving only the anterior curved part in the individuals showing the strongest phenotype (Fig. 1D, E). Since the mouth hooks are necessary to open the operculum of the egg, this may explain the failure of the egg hatching. With lower penetrance, the whole cephalopharyngeal skeleton showed a deformed structure probably due to a less sclerotized structure, especially the median tooth, the H-piece and the lateralgräten (fig. 1D, E). These data demonstrate that the formation of the sclerotized parts of the head skeleton is sensible to high level of DmKal-1 protein. There is a perfect correlation between the expression pattern of the *179y*-GAL4 driver in the cephalopharyngeal skeleton and the components of the head skeleton, which show a reduced sclerotization.

To prove the specificity of the head skeleton phenotype induced by *kal-1 *expression, we tested if the expression of the *Neuroglian *form *Nrg*^180 ^[26], which contains five FnIII domains, was able to induce a phenotype similar to that produced by *kal-1*. The Neuroglian expressing larvae were viable and able to hatch and did not show cephalopharyngeal skeleton alterations (data not shown), suggesting that a simple increase in FnIII containing proteins in the matrix does not induce alterations of the head skeleton. Taken together with the expression profile of *kal-1 *in the mandibular segment, these data suggest an involvement of *kal-1 *in some aspects of the formation of the head skeleton.

### Structure-function dissection of DmKal-1

**Figure 3:**
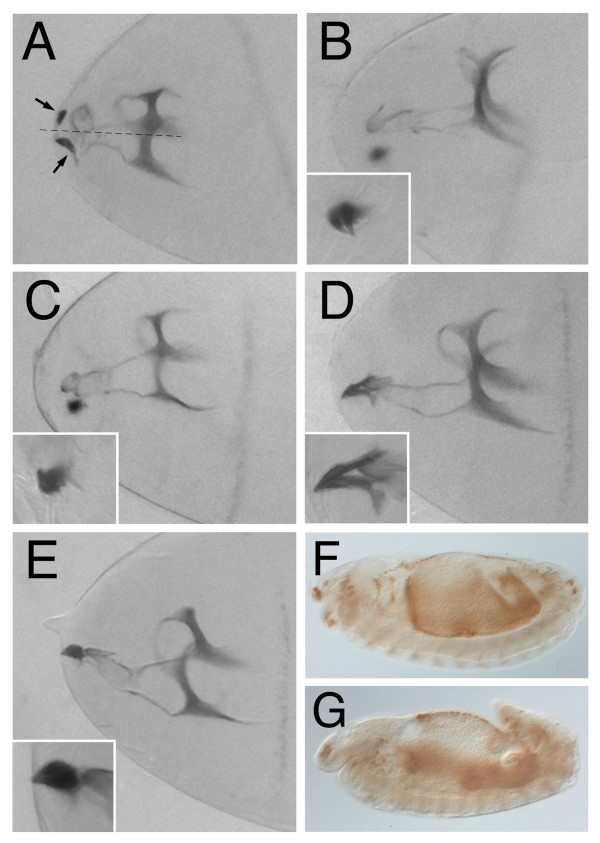
**Cephalopharyngeal skeleton phenotypes induced by overexpression of the different DmKal-1 mutant proteins**. (A, B, C, D, E) Head cuticle of stage 17 embryos. (A) Stage 17 *179y*-Gal4/+; UAS-*kal-1 [N236K] *embryo. The head skeleton structure appears less sclerotized and the median tooth is altered. Moreover, the mouth hooks (arrows) lack the posterior part and appear distant from the mid-line (dotted line). (B) Stage 17 *179y*-Gal4/+; UAS-*kal-1 [C127/128S] *embryo. (C) Stage 17 *179y*-Gal4/+; UAS-*kal-1 [C127/128S+N236K] *embryo. (D) Stage 17 *179y*-Gal4/+; UAS-*kal-1 [C85S] *embryo. The mouth hooks base shows a reduction of the dorsal process. (E) Stage 17 *179y*-Gal4/+; UAS-*kal-1 [R159T+R161L] *embryo. In the insets, a magnification of the hooks. (F, G)) Stage 13 embryos immunostained with anti-Hind antibody. (F) *179y*-Gal4/+; UAS-*kal-1*; UAS-*kal-1 *embryo. GBR occurs normally. (G) *179y*-Gal4/+; UAS-*kal-1 [N236K] *embryo. The embryo shows an incomplete GBR process.

By using a neuronal phenotype induced by overexpression, it was shown that the *C. elegans *Kal-1 (CeKal-1) WAP and FnIII domains seem to mediate different functions [12]. We therefore decided to find out which domains of DmKal-1 were required for the head skeleton phenotype described above. A C-terminal truncated CeKal-1 protein, missing part of the first FnIII domain and all the other FnIII domains, did not induce any phenotype when expressed in AIY interneurons [12]. We reproduced this deletion (Fig. 2B), found in some human KS patients [20], and the UAS-*kal-1 [W200-STOP] *construct was expressed using the *179y*-GAL4 driver. All the lines analyzed did not show any phenotype (data not shown). We then reproduced another human mutation (nonsense mutation at Q_421_) [20], which causes in *Drosophila *the deletion of the C-terminal end of the protein, just after the second FnIII repeat (Fig. 2C). The deleted sequence contains a predicted heparan-sulfate binding site (P^441^HKEKV^446^). Also in this case, the expression of UAS-*kal-1 [H384-STOP]*, driven by *179y*-GAL4, did not induce any phenotype (data not shown), indicating that the C-terminal region is necessary for the function of DmKal-1. We can not exclude, however, that the deletion of the C-terminal domain(s) may alter the stability of the protein. We then tested a specific point mutation in the first FnIII repeat, which mimics a mutation at an equivalent position identified in a human KS patient  (Fig. 2D)[20]. In *C. elegans *the same missense mutation abrogates heparin dependent cell adhesion and the ability of the protein to induce cell axonal branching [12]. In addition, it was also demonstrated that an equivalent mutation in the human anosmin-1 affects the chemiomigration activity of the protein on GN11 cells [19]. When expressed with the *179y*-GAL4 driver, the UAS-*kal-1 [N236K] *construct produced profound head alterations, including abnormal median tooth development as well as mouth hook defects and a skeleton with a less sclerotized structure. Surprisingly, the Dmkal-1 [N236K] protein also induced HI defects (Fig. 3A). This mutant protein retained the ability to induce the defects produced by the wild type DmKal-1 protein (Fig. 1E), but also altered the HI process, suggesting that it has an additional activity in respect to the wild type protein. Four different DmKal-1 [N236] lines were analyzed, and all of them showed these phenotypes (Table 1). There was a correlation between lethality penetrance and the number of larvae showing HI phenotypes. We can exclude that the protein level of DmKal-1 [N236] is higher than those of the wild type because even DmKal-1 lines producing high lethality did not shown any HI phenotypes (Table 1). Furthermore, all the other mutant lines affecting the other domains never showed HI defects (data not shown). Analyzing the *UAS-kal-1 [N236K] *lines that gave the strongest HI phenotypes, we observed that a significant part of the embryos displayed an incomplete GBR (Fig. 3F, G). Since *179y*-GAL4 also drives the expression in the amnioserosa, this phenotype might be due to altered adhesion between amnioserosa and germ band cells. The GBR phenotype was not induced by the expression of the wild type protein, also when we used the P [UAS-*kal-1*]^II and III ^line (Fig. 3F).

**Table 1:** Lethality and HI defects in DmKal-1 and DmKal-1 [N236K] larvae.

transformant lines	lethality (number)	HI defects
DmKal-1 (12)	31% (139)	0%
DmKal-1 (30)	32% (198)	0%
DmKal-1 (47)	43% (226)	0%
DmKal-1 (II and III)	72% (193)	0%

DmKal-1 [N236] (12A)	28% (230)	9%
DmKal-1 [N236] (13)	80% (123)	24%
DmKal-1 [N236] (10B)	85% (192)	22%
DmKal-1 [N236] (1B)	86% (102)	37%

To determine whether the WAP domain was essential for the induction of the head skeleton phenotype and for the alterations in the HI process induced by the N236K substitution, we introduced a double substitution C-S in the four-disulfide core motif both in the wild type protein (UAS-*kal-1 [C127/128S]*) (Fig. 2E) and in the DmKal-1 [N236K] mutant protein (UAS-*kal-1 [C127/128S+N236K]*) (Fig. 2F). The C127/128S substitution as well as the C127/128S+N236K combination induced alterations of the head skeleton similar to the wild type DmKal-1 protein (Fig. 3B, C). This demonstrates that the double substitution in the WAP domain does not alter the ability of DmKal-1 to induce head skeleton phenotypes. On the other hand, the alteration of the WAP domain suppresses the HI phenotype induced by the N236K mutation. These experiments suggest that, like in *C. elegans*, the *Drosophila *Kal-1 WAP domain is not essential for some functions of the protein but that, nevertheless, the function of the fibronectins and of the WAP domain is strictly interdependent, at least in this context.

At the N-terminus region of the DmKal-1 protein there is a conserved CR region, containing four out of the eight cysteines residues found in the human protein. We prepared a UAS construct with the C-S substitution at position 85 (UAS-*kal-1 [C85S]*) (Fig. 2G) to test the function of this domain. We analyzed five different transgenic lines but only one showed reduced viability and delay of hatching when crossed with *179y*-GAL4, while the others did not show any appreciable phenotype. The head skeleton showed a thin structure, while the mouth hooks base showed only a reduction of the dorsal process (Fig. 3D). Increasing the UAS-*kal-1 [C85S] *copy number, we registered a small increase of the lethality, even though the cephalopharyngeal skeleton phenotype was not as severe as the phenotype induced by the wild type *kal-1 *gene. We can formulate at least two possible explanations for this result: it may depend on a lower activity of the DmKal-1 [C85S] protein or on a lower amount of the mutant protein produced in all the lines examined. Since we do not have a specific antibody against Dmkal-1 we can not exclude the second possibility. Anyway, it is worth noting that a CR mutated form of DmKal-1 retains a significant activity, at least in respect to the phenotype analyzed.

### Function of the fibronectins and of a predicted heparan-sulfate binding site in the adhesion property and activity of DmKal-1

**Figure 4:**
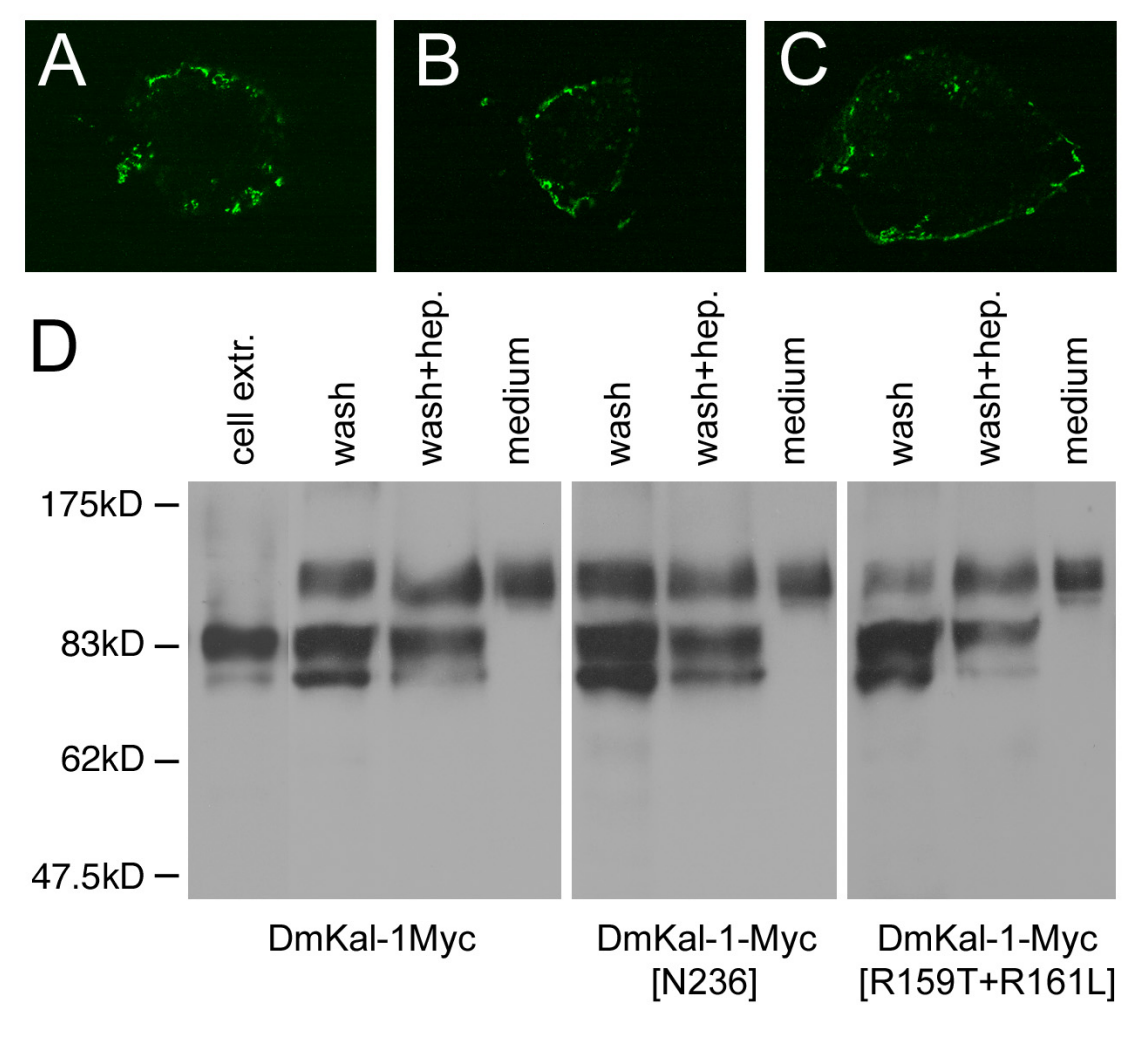
**Cellular localization and western blot analysis of wild type and fibronectin mutant forms of DmKal-1-Myc in COS-7 transfected cells**. (A, B, C) Confocal analysis on unpermeabilized cells. DmKal-1-Myc proteins were detected by monoclonal 9E10 antibody. (A) DmKal-1-Myc is secreted from the transfected cells and bind to the cell surface. (B) DmKal-1-Myc [N236K] localizes on the cell surface of the transfected cells. (C) Dmkal-1-Myc [R159T+R161L] localizes on the cell surface of the transfected cells. (D) Western blot analysis of recombinant Dmkal-1-Myc proteins performed using the polyclonal ab9106-100 antibody. Recombinant DmKal-1-Myc proteins present in the cell extracts (cell extr.), in the wash with fresh medium (wash), in the wash with 100 μg/ml heparin (wash + hep.), and in the conditioned medium prior of the washes (medium). The intensity of the lower band varied in different experiments.

It is striking that the N236K mutation promotes additional phenotypes that are not induced by the wild type protein, while in *C. elegans *the same mutation produces a partial loss of function [12]. Since the fibronectin mutatedCeKal-1 protein looses the heparin-dependent cell adhesion property of wild type CeKal-1, we decided to test if the cell-binding activity of DmKal-1 was abolished by the N236K substitution. First, we analyzed the cell adhesion property of the wild type DmKal-1 protein, transiently expressing a myc-tagged form of DmKal-1 in COS-7 cells. We performed immunofluorescence analysis on unpermeabilized cells, using a monoclonal anti c-myc antibody (9E10). As shown in Fig. 4A, DmKal-1-Myc localized at the cell surface of the transfected cells, confirming that also the *Drosophila *ortholog of anosmin-1 is secreted in the extracellular space and is able to bind to the outer side of the cell membrane. Western blot analysis of cell extracts revealed the presence of two bands, representing different modified forms of Dmkal-1 (Fig. 4D). In fact the molecular weight of the two bands exceeds the theoretical weight of 60 kD of DmKal-1-Myc. We found that DmKal-1-Myc was abundant in the medium (Fig. 4D and material and methods), where only one isoform of an even higher molecular weight was detected, suggesting that the protein is further modified. The DmKal-1-Myc protein was released from the cell surface when the cells were incubated in fresh medium for 30 min (Fig. 4D), indicating a weak binding of DmKal-1-Myc to the cell surface. When the cells were further incubated for 30 min in a medium containing 100 μg/ml of heparin, an additional small amount of DmKal-1-Myc protein was released from the cell surface (Fig. 4D). All the three forms of the DmKal-1-Myc protein can be detected on the cell surface, indicating that the further modification of the protein occurs on the outer side of the cell membrane.

**Figure 5:**
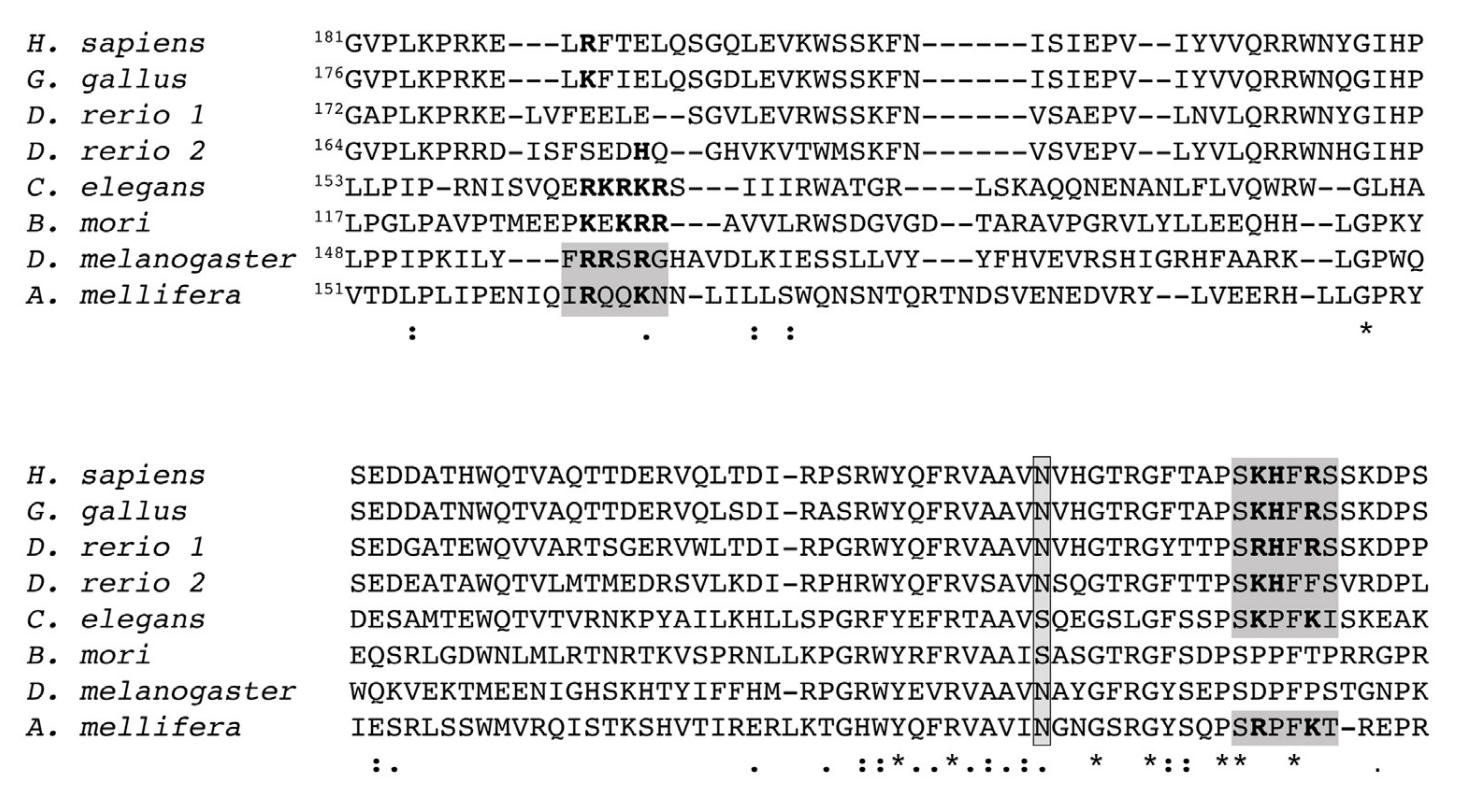
**Evolutionary conservation of the heparan-sulfate binding site of the first FnIII domain**. Amino acid sequence alignment of the first FnIII domain of different anosmin-1 proteins. An heparan-sulfate binding motif is conserved in the C-terminal part of the domain in all vertebrates, in *C. elegans *and in *A. mellifera*. In *D. melanogaster*, a complete heparan-sulfate binding motif is present at the N-terminus while, in *A. mellifera*, only a partially conserved motif is present in this region. In bold, the basic residues inside the motif. In the box, the asparagine at position 267 of the human protein and the equivalent amino acid in the other species.

Like DmKal-1-Myc also DmKal-1-Myc [N236K] showed a cell surface localization in transfected COS-7 cells (Fig. 4B). The mutant protein was released in the fresh medium and in the heparin-containing medium with a pattern that was identical to the wild type protein (Fig. 4D). This suggested that the N236K substitution does not disrupt DmKal-1 interaction with the cell surface. In contrast, the equivalent mutation in *C. elegans *completely abolishes the interaction of CeKal-1 with the cell membrane [12]. These results might explain why DmKal-1 [N236K] maintains its biological activity *in vivo*. A possible structural basis for the different behavior of the two proteins can be found when comparing the sequence of the first fibronectin of anosmin-1 in different species (Fig. 5). In all the vertebrates and in some of the invertebrates, there is a conserved heparan-sulfate binding site consensus (XBBXBX where B is a basic residue and X any other one) near to the site of the mutation [27]. This sequence is present also in *C. elegans*, even if it is only partially conserved. In *D. melanogaster*, as in *B. mori*, this heparan-sulfate binding site is absent. A complete consensus sequence for a heparan-sulfate binding site is instead present at the N-terminus of the first *Drosophila *fibronectin (Fig. 5).

We then hypothesized that the removal of the predicted heparan-sulfate binding site might inactivate the protein. We changed the F^157^RRSRG^162 ^site to an F^157^RTSLG^162^, substituting two basic residues. We tested if this protein retained the binding property to the cell surface, transfecting COS-7 cells with a Myc tagged form of DmKal-1 [R159T+R161L] (Fig. 2H). We found that the mutant protein still localized to the cell surface (Fig. 4C), with a distribution pattern similar to the wild type protein (Fig. 4D). We also created transgenic lines bearing a UAS-*kal-1 [R159T+R161L] *construct. *179y*-GAL4 driven expression of this mutant protein induced lethality, and delay of hatching as the wild type DmKal-1 protein (data not shown). The head skeleton also showed the same defects as those induced by the wild type protein (Fig. 3E). These observations indicate that the destruction of this heparan-sulfate binding site consensus neither affects the binding to the cell surface nor the *in vivo *activity of the protein. A possible explanation might be the redundancy of heparan-sulfate binding sites, but another possibility is the dispensability of this site in the particular context in which the protein has been tested.

### Effects of mutant DmKal-1 proteins on wing development

**Figure 6:**
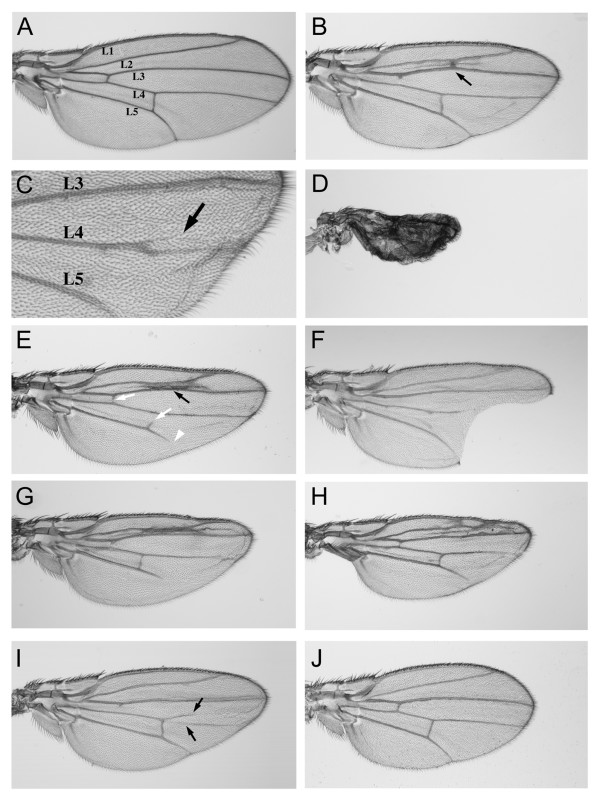
**Wing phenotypes induced by overexpression of wild type and mutant DmKal-1 proteins in the wing disc**. (A, B, E, G, H) Wings derived from flies emerged at 18°C. (A) *MS1096*-GAL4/+ control wing with designations of longitudinal veins (L1-L5). (B) *MS1096*-Gal4/+; UAS-*kal-1*/+; UAS-*kal-1*/+ wing shows a slightly shorter proximal-distal axis with a reduction of the L2-L3 intervein territory and a partial fusion of the L2 and L3 veins (black arrow). (C) Higher magnification of an *MS1096*-Gal4/+; UAS-*kal-1*/+ wing (25°C), with splitting of the L4 vein into anterior and posterior branches. The arrow indicates the anterior branch. (D) At 25°C, *MS1096*-Gal4/+; UAS-*kal-1*/+; UAS-*kal-1*/+ flies show a stronger phenotype with an extremely reduced wing. (E) *MS1096*-Gal4/+; UAS-*kal-1 [N236K]/+ *wing, which shows a partial fusion of the L2 and L3 veins (black arrow). The break of the anterior and posterior cross-veins (white arrows) and the reduction of the distal L5 vein (white arrowhead) are also found in the control. (F) At 25°C, *MS1096*-Gal4/+; UAS-*kal-1 [R159T+R161L]*/+ wing shows a notched edge between L3-L5 veins. (G) *MS1096*-Gal4/+; UAS-*kal-1 [C127/128S]*/+ wing. (H) *MS1096*-Gal4/+; UAS-*kal-1 [C127/128S+N236K]*/+ wing. (I) At 25°C, *MS1096*-Gal4/+; UAS-*kal-1 [C85S]*/+ wing shows a proximal-distal reduction and the splitting of the L4 vein (arrows). (J) At 25°C, *MS1096*-Gal4/+; UAS-*Nrg180*/+ wing shows a proximal-distal shortening. All wings are from females and they are oriented with anterior upwards and proximal to the left.

Finally, we decided to test whether the effects of the mutant DmKal-1 proteins might be reproduced in a different tissue. The data obtained by overexpression in the head skeleton suggested that the activity of the different DmKal-1 domains are, at least in part, dispensable. The dispensability of the WAP domain in some context has been hypothesized to explain the case of a patient with the C163Y mutation in the WAP domain, who showed a relatively well conserved sexual phenotype and hormone levels [28]. We chose the wing disc, because alterations in the developmental program of this organ are easily detectable in adult flies. We decided to use the *MS1096*-GAL4 driver [29] to express the wild type form of DmKal-1 in the whole wing disc. We detected a variety of defects, which were depending on the expression level and therefore modified by increasing the temperature or the copy number. Because the high variability in the intensity of the different phenotypes among the different lines of each construct, we took in account only qualitative differences. At low temperature (18°C), P [UAS-*kal-1*] transgenic lines did not show any appreciable phenotype (data not shown), while the P [UAS-*kal-1*]^II and III ^line showed a slight reduction of the proximal-distal (P-D) axis of the wing and a reduction of the L2-L3 intervein territory, often leading to the fusion of the two veins (Fig. 6A, B). We also found an appreciable lethality during the pupal stage. At 25°C, P [UAS-*kal-1*] transgenic lines showed splitting of the L4 vein into anterior and posterior branches with low penetrance (Fig. 6C). At the same temperature (25°C), the P [UAS-*kal-1*]^II and III ^line showed a high lethality with only some escapers showing a dramatic reduction of the whole wing (Fig. 6D). The specificity of these phenotypes was also in this case tested expressing *Nrg*^180 ^in the wing disc. At 25°C, we detected a reduction of the P-D axis of the wing (Fig. 6J) suggesting that this alteration may depend on an unspecific effect of the fibronectin domains. On the other hand, we did not find any reduction of intervein territory and or splitting/fusion of veins, suggesting that these phenotypes are specifically related to the expression of DmKal-1.

We then tested all the Dmkal-1 mutant forms. The two deleted proteins, one lacking all the fibronectins (W200-STOP) and the other one truncated in the C-terminal region (H384-STOP), did not induce any phenotypes also at 28°C (data not shown), confirming that the missing parts are indispensable for the function or for the stability of the protein. DmKal-1 [N236K] induced a phenotype similar to the wild type form (Fig. 6E). Same lines showed high lethality and strong reduction of the whole wing also at 18°C (data not shown). In this organ, however, we did not find appreciable qualitative differences in respect to that induced by the wild type protein. This indicated that, in the context of the wing, the N236K substitution does not confer to the protein the ability to induce new phenotypes. At 18°C, DmKal-1 [R159T+R161L] induced only a small reduction of the P-D axis, with no other visible defects (data not shown). At 25°C, we found a stronger reduction of the P-D axis and, frequently, splitting of the L4 vein (data not shown). Reduction of the L2-L3 intervein region was only occasionally found, while we detected with higher frequency wings showing the edge between L3 and L5 notched, (Fig. 6F). We never found this phenotype when we expressed a single dose of wild type or other mutant DmKal-1 proteins, but we found this phenotype with low penetrance when we expressed P [UAS-*kal-1*]^II and III ^at 18°C (data not shown). The expression of DmKal-1 [C127/128S] and DmKal-1 [C127/128S+N236K] induced the same phenotypes as the wild type protein (Fig. 6G, H), suggesting that the activity of the WAP domain is dispensable also in this context. The expression of Dmkal-1 [C85S] at 18°C and at 25°C caused milder phenotypes, but often we found a broad splitting of the L4 vein (Fig. 6I).

## Discussion

In the present work, we report the first functional characterization of DmKal-1. We show that DmKal-1, like its orthologs analyzed until now, is secreted in the extracellular space where it localizes on the cell surface and, in greater amount, accumulates in the medium (Fig. 4A, 4D). Dmkal-1 is detected on the cell surface of transfected COS7 cells, but this binding appears particularly weak, since the protein is easily released after simple substitution of the medium. Only a small amount of protein is further released washing the cells or after addition of heparin (Fig. 4D). DmKal-1 has less FnIII domains in respect to the human ortholog, and this may explain the weakness of the binding to the cell surface.

Using an overexpression approach, we also demonstrate that DmKal-1 is able to alter the cephalopharyngeal skeleton formation (Fig. 1D, E) and to determine strong defects during wing development (Fig. 6A, B, C, D). Interestingly, there is a tight correlation between the head skeleton phenotype induced by *kal-1 *overexpression and the wild type *kal-1 *expression profile in the gnathal region. In *C. elegans*, a CeKal-1 loss of function mutation as well as CeKal-1 overexpression produce ventral closure and male tail defects [11]. These defects are due to the disruption of correct cell contacts and shapes during epithelial morphogenesis. We propose that incorrect contacts among cells may be responsible for incorrect secretion of the chitinous material necessary for the formation of the head skeleton, when DmKal-1 is overexpressed. A *kal-1 *mutant allele will be necessary to confirm this hypothesis.

The elimination of all the FnIII repeats produces a protein completely lacking activity *in vivo *[12] (and present study). We have also shown that the elimination of the C-terminal region of DmKal-1, which contains a putative heparan-sulfate binding site, completely suppresses the activity of the protein. It has been showed that the reduction of the number of FnIII repeats determines a weaker cell surface association and a lower heparan-sulfate binding affinity [30]. We can not exclude, however, that the Drosophila Kal-1 protein looses its stability when these deletions are produced. We need to obtain a specific antibody to exclude this possibility. The N236K mutation in the first FnIII domain of DmKal-1, which mimics a mutation at an equivalent position identified in a human KS patient [20], produces additional phenotypes compared to the wild type protein. In fact, the expression of this mutated form of DmKal-1 has a stronger effect and also induces defects in HI and GBR processes (Fig. 3A, G). It is also interesting to note that, in the wing, DmKal-1 [N236K] overexpression is instead similar to that of the wild type protein (Fig. 6B, E). This suggests that the function of the FnIII domains change in respect to the tissue under examination. The binding activity of DmKal-1 is not impaired by the N236K substitution (Fig. 4B, D), and this is different to the effects produced by an equivalent mutation introduced in CeKal-1 [12]. In *C. elegans*, there is a tight correlation between the loss of adhesion and the partial loss of function of the protein. We can, however, exclude that the different phenotype depends on the different location of the heparan-sulfate binding site in the first FnIII repeat. In fact, the elimination of the heparan-sulfate binding site consensus in DmKal-1 does not induce a loss of adhesive property (Fig. 4C, D) and a loss of function *in vivo *(Fig. 3E and 6F). Two new missense mutations were found in the first FnIII repeat (V263G and R262P) that causes the Kallmann's Syndrome, confirming the importance of this region for the function of the protein [30,31]. Nevertheless, these mutations do not completely impair the function of the protein [12], and may also confer new activities in some contexts (Fig. 3A, G). It is interesting to note that the elimination of the heparan-sulfate binding site consensus in the first FnIII repeat modifies the activity of the protein in the wing disc but not in the cephalopharyngeal skeleton, again emphasizing the relevance of the extracellular context for the protein activity.

Mutations in the CR region or in the WAP domain did not cause loss of function of DmKal-1 in respect to its ability to induce head skeleton phenotypes (Fig. 3B, D) or to alter wing development (Fig. 6G, I). Even if our data suggest that the cysteine-serine substitution in the CR region might cause a reduction in DmKal-1 protein activity, the overexpression of both DmKal-1 [C85S] and DmKal-1 [C127/128S] proteins results in phenotypes similar to the wild type. A C163Y substitution in the WAP domain was found in one patient affected by KS in which the sexual phenotype and the sexual hormone levels were well conserved [28]. It has been suggested that the WAP domain is dispensable for some of the biological activities of the KAL-1 protein, at least in the GnRH production. Our data are in agreement with this hypothesis.

The double substitution C127/128S+N236K, in which the additional phenotype induced by the N236K substitution is suppressed by the mutation of the WAP domain (Fig. 3C), indicates interdependence between the function of the WAP domain and of the FnIII repeats. This suggests that the function of the WAP domain is in some way linked to the function of the fibronectins. It is possible to hypothesize that the WAP domain, and possibly the CR region, has a regulatory role in respect to the fibronectins. We envision that the WAP domain, but also the CR region, may interfere with the adhesive property of the FnIII repeats, facilitating or preventing the interaction with heparn-sulfates or other interacting molecules Consistent with this hypothesis is the finding that the mutation of the WAP domain of CeKal-1 abrogated the heparan-dependent binding activity of the protein [12]. On the other hand, the C172R substitution in the WAP domain of the human ortholog does not alter the heparan-sulfate binding characteristics of the protein [30]. Even though these data appear contradictory, it is possible to imagine that the adhesive property of anosmin-1 depends also on the extracellular matrix composition that is in turn different in diverse tissues. The adhesive property of anosmin-1 to the cell surface may change in different cells, as well as the contribution of the WAP domain and of the CR region may therefore be essential in some extracellular matrix contexts but nonessential in others.

## Conclusion

Overexpression of proteins bearing point mutations that alters the different domain of DmKal-1 in two different tissue, the cephalopharyngeal skeleton of the embryo and the wing disc, suggests that the relevance of each domain is dependent on the extracellular contexts. We suggest that FnIII repeats have the main role for the activity of the protein, while the CS region and the WAP domain have only a modulator role. These data may help to understand the effect of point mutations affecting the different domains of KAL-1 and that cause complex phenotypes in KS affected patients.

## Methods

### Fly stocks

*Drosophila melanogaster *stocks were maintained at 25°C on standard medium. *y, w*^67*c*23 ^has been used as control and also for the production of the transgenic lines carrying the different constructs. Several independent lines of each pUAST construct were obtained by P-element germ-line transformation [33] and at least tree different lines were analyzed for the wild type and each mutant. The *179y*-GAL4 and UAS-*GFP *lines were obtained from Bloomington Stock Center. UAS-*Nrg*^180 ^stock was gently provided by Luis Garcia-Alonso, and the *MS1096*-GAL4 line by Daniela Grifoni.

### *In situ *hybridization and immunohistochemistry

*In situ *hybridization and immunohistochemistry experiments were performed with minor modifications as described by [34]. Antisense DIG labeled RNA, corresponding to the full-length cDNA, was used as a probe. For the immunohistochemistry experiments, anti-Hind 1G9 (Developmental Studies Hybridoma Bank) mouse monoclonal antibodies were used at 1:100.

### Generation of *kal-1 *and *kal-1-myc *constructs

Mutated forms of *kal-1 *and *kal-1-myc *were produced by the QuikChange Site-Directed mutagenesis Kit (Stratagene), using appropriated oligonucleotide primers. Full coding region of *kal-1 *cDNA and mutated forms of *kal-1 *were inserted into the *Eco*RI-*Xho*I restriction sites of the pUAST tranforming vector [24]. *kal-1-myc *was obtained cloning a PCR product in into the *Eco*RI-*Sal*I sites of the pMT21 transformation vector containing the c-myc epitope [35]. Primer sequences are available from the authors upon request.

### Preparation of embryonic cuticle and fly wings

*179y*-GAL4 flies were crossed with the different UAS lines at 28°C. For the determination of the lethality, stage 17 embryos were collected and counted after 48 hours. Embryos were dechorionated in 50% bleach and counted. For the morphological analysis, after dechorionation, the embryos were mounted in Hoyer's solution cleared for several days at 65°C [36]. Adult wings were dissected and dehydrated in ethanol, mounted in lactic acid/ethanol (6:5) [37], and examined under light microscope.

### Cell culture

Monkey kidney COS-7 cells were maintained in exponential growth in Dulbecco's modified Eagle's medium (DMEM, Highclone) containing 10% fetal bovine serum (Highclone). All constructs were transfected using Polyfect (Qiagen) according to instructions provided by the manufacturer.

### Transfection experiments and western blotting

24 hours after transfection, the serum-containing medium was replaced with serum-free medium. After 48 hours, the medium (4 ml) was recovered and the cells were washed in PBS and left in serum-free medium for 30 min and in serum-free medium containing 100 μg/ml heparin (sodium salt) for further 30 min. The medium of the first wash and the heparin-containing medium were concentrated by Centricon YM10 (Millipore, Milano, Italy). After recovery of the heparin-containing medium, the proteins were extracted from the cells. Cells were lysed in 50 mM Tris pH 8, 300 mM NaCl, 1% Triton X-100, supplemented with protease inhibitors (SIGMA). Samples were subjected to a brief sonication (3 × 15s) and then centrifuged at 10000 g for 10 min at 4°C to remove cellular debris. The protein samples were resuspended in SDS sample buffer (20 mMTris-HCl pH 6.8, 2% SDS, 5% β-mercaptoethanol, 2.5% glycerol and 2.5% bromophenol blue). We used the whole sample for the washes with fresh medium and for the washes with medium containing 100 μg/ml heparin, while we used 1/200 of the growth medium. The samples were subjected to standard SDS-PAGE electrophoresis followed by transfer to a polyvinylidene difluoriode membrane (PVDF, Amersham). We used the anti-myc tag antibody ab9106-100 (abcam) at 1:1000. This antibody did not reveal any signal in non-transfected cells. Visualization of antibody binding was carried out with the enhanced chemoluminescence (ECL PLUS) reagent according to the manufacturer's protocol (Amersham). Horseradish peroxidase (HRP)-conjugated antibodies were from Amersham Pharmacia (1:5000).

### Immunofluorescence

For immunofluorescence experiments, cells were grown and transfected on coverslips. 48 hours after transfection, cells were blocked in PBS containing 10% pig serum for 10 min and incubated with the monoclonal anti c-myc (9E10) supernatant for 1 h at 37°C. Cells were then fixed for 10 min with a solution of 4% paraformaldehyde in PBS, and incubated with FITC anti-mouse Ig (DAKO) at 1:200 for 1 h at RT. Coverslips were mounted with Vectashield (DBA) and examinated with a confocal microscope (Biorad).

## Authors' contributions

DA performed the characterization of embryonic and wing phenotypes; prepared part of the constructs injected in Drosophila; drafted the manuscript, with the contribution of FG and EIR. MRG performed Drosophila embryonic injections and the preparation of the transgenic lines with the contribution of DA and VP. VP performed part of the constructs injected in Drosophila. EIR and MRG prepared the constructs for the *in vitro *cell experiments. *In vitro *cell analysis was performed by ER and DA in collaboration with MRG. FG oversaw the project. All authors reviewed and approved the final manuscript.

## References

[B1] Kallmann F, Schoenfeld WA, Barrera SE (1944). The genetic aspects of primary eunuchoidism. Am J Ment Defic.

[B2] Schwanzel-Fukuda M, Pfaff DW (1989). Origin of luteinizing hormone-releasing hormone neurons. Nature.

[B3] Wray S, Grant P, Gainer H (1989). Evidence that cells expressing luteinizing hormone-releasing hormone mRNA in the mouse are derived from progenitor cells in the olfactory placode. Proc Natl Acad Sci USA.

[B4] Hardelin JP, Julliard AK, Moniot B, Soussi-Yanicostas N, Verney C, Schwanzel-Fukuda M, Ayer-Le Lievre C, Petit C (1999). Anosmin-1 is a regionally restricted component of basement membranes and interstitial matrices during organogenesis: implications for the developmental anomalies of X chromosome-linked Kallmann syndrome. Dev Dyn.

[B5] Colquhoun-Kerr JS, Gu WX, Jameson JL, Withers S, Bode HH (1999). X-linked Kallmann syndrome and renal agenesis occurring together and independently in a large Australian family. Am J Med Genet.

[B6] Hermanussen M, Sippell WJ (1985). Heterogeneity of Kallmann's syndrome. Clin Genet.

[B7] Wegenke JD, Uehling DT, Wear JB, Gordon ES, Bargman JG, Deacon JS, Herrmann JP, Opitz JM (1975). Familial Kallmann syndrome with unilateral renal aplasia. Clin Genet.

[B8] Franco B, Guioli S, Pragliola A, Incerti B, Bardoni B, Tonlorenzi R, Carrozzo R, Maestrini E, Pieretti M, Taillon-Miller P, Brown CJ, Willard HF, Lawrence C, Persico MG, Camerino G, Ballabio A (1991). A gene deleted in Kallmann's syndrome shares homology with neural cell adhesion and axonal path-finding molecules. Nature.

[B9] Legouis R, Hardelin JP, Levilliers J, Claverie JM, Compain S, Wunderle V, Millasseau P, Le Paslier D, Cohen D, Caterina D, Bougueleret L, Delemarre-Van de Waal H, Lutfalla G, Weissenbach J, Petit C (1991). The candidate gene for the X-linked Kallmann syndrome encodes a protein related to adhesion molecules. Cell.

[B10] Dode C, Levilliers J, Dupont JM, De Paepe A, Le Du N, Soussi-Yanicostas N, Coimbra RS, Delmaghani S, Compain-Nouaille S, Baverel F, Pecheux C, Le Tessier D, Cruaud C, Delpech M, Speleman F, Vermeulen S, Amalfitano A, Bachelot Y, Bouchard P, Cabrol S, Carel JC, Delemarre-van de Waal H, Goulet-Salmon B, Kottler ML, Richard O, Sanchez-Franco F, Saura R, Young J, Petit C, Hardelin JP (2003). Loss-of-function mutations in *FGFR1 *cause autosomal dominant Kallmann syndrome. Nat Genet.

[B11] Rugarli EI, Di Schiavi E, Hilliard MA, Arbucci S, Ghezzi C, Facciolli A, Coppola G, Ballabio A, Bazzicalupo P (2002). The *Kallmann syndrome *gene homolog in *C. elegans *is involved in epidermal morphogenesis and neurite branching. Development.

[B12] Bülow HE, Berry KL, Topper LH, Peles E, Hobert O (2002). Heparan sulfate proteoglycan-dependent induction of axon branching and axon misrouting by the Kallmann syndrome gene *kal-1*. Proc Natl Acad Sci USA.

[B13] Andrenacci D, Le Bras S, Grimaldi MR, Rugarli EI, Graziani F (2004). Embryonic expression pattern of the Drosophila Kallmann syndrome gene *kal-1*. Gene Expr Patterns.

[B14] Legouis R, Lievre CA, Leibovici M, Lapointe F, Petit C (1993). Expression of the *KAL *gene in multiple neuronal sites during chicken development. Proc Natl Acad Sci USA.

[B15] Rugarli EI, Lutz B, Kuratani SC, Wawersik S, Borsani G, Ballabio A, Eichele G (1993). Expression pattern of the *Kallmann syndrome *gene in the olfactory system suggests a role in neuronal targeting. Nat Genet.

[B16] Lutz B, Kuratani S, Rugarli EI, Wawersik S, Wong C, Bieber FR, Ballabio A, Eichele G (1994). Expression of the *Kallmann syndrome *gene in human fetal brain and in the manipulated chick embryo. Hum Mol Genet.

[B17] Soussi-Yanicostas N, Faivre-Sarrailh C, Hardelin JP, Levilliers J, Rougon G, Petit C (1998). Anosmin-1 underlying the X chromosome-linked Kallmann syndrome is an adhesion molecule that can modulate neurite growth in a cell-type specific manner. J Cell Sci.

[B18] Soussi-Yanicostas N, De Castro F, Julliard AK, Perfettini I, Chedotal A, Petit C (2002). Anosmin-1, defective in the X-linked form of Kallmann syndrome, promotes axonal branch formation from olfactory bulb output neurons. Cell.

[B19] Cariboni A, Pimpinelli F, Colamarino S, Zaninetti R, Piccolella M, Rumio C, Piva F, Rugarli EI, Maggi R (2004). The product of X-linked Kallmann's syndrome gene (KAL1) affects the migratory activity of gonadotropin-releasing hormone (GnRH)-producing neurons. Hum Mol Genet.

[B20] Hardelin JP, Levilliers J, Blanchard S, Carel JC, Leutenegger M, Pinard-Bertelletto JP, Bouloux P, Petit C (1993). Heterogeneity in the mutations responsible for X chromosome-linked Kallmann syndrome. Hum Mol Genet.

[B21] Maccoll G, Bouloux P, Quinton R (2002). Kallmann syndrome: adhesion, afferents, and anosmia. Neuron.

[B22] DeFalco TJ, Verney G, Jenkins AB, McCaffery JM, Russell S, Van Doren M (2003). Sex-specific apoptosis regulates sexual dimorphism in the *Drosophila *embryonic gonad. Dev Cell.

[B23] Mohler J, Mahaffey JW, Deutsch E, Vani K (1995). Control of *Drosophila head *segment identity by the bZIP homeotic gene *cnc*. Development.

[B24] Brand AH, Perrimon N (1993). Targeted gene expression as a means of altering cell fates and generating dominant phenotypes. Development.

[B25] Manseau L, Baradaran A, Brower D, Budhu A, Elefant F, Phan H, Philp AV, Yang M, Glover D, Kaiser K, Palter K, Selleck S (1997). GAL4 enhancer traps expressed in the embryo, larval brain, imaginal discs, and ovary of *Drosophila*. Dev Dynamics.

[B26] Garcia-Alonso L, Romani S, Jimenez F (2000). The EGF and FGF receptors mediate *neuroglian *function to control growth cone decisions during sensory axon guidance in Drosophila. Neuron.

[B27] Hileman RE, Fromm JR, Weiler JM, Linhardt RJ (1998). Glycosaminoglycan-protein interactions: definition of consensus sites in glycosaminoglycan binding proteins. Bioessays.

[B28] Sato N, Katsumata N, Kagami M, Hasegawa T, Hori N, Kawakita S, Minowada S, Shimotsuka A, Shishiba Y, Yokozawa M, Yasuda T, Nagasaki K, Hasegawa D, Hasegawa Y, Tachibana K, Naiki Y, Horikawa R, Tanaka T, Ogata T (2004). Clinical assessment and mutation analysis of *Kallmann syndrome 1 *(*KAL1*) and *fibroblast growth factor receptor 1 (FGFR1, or AL2) *in five families and 18 sporadic patients. J Clin Endocrinol Metab.

[B29] Capdevila J, Guerrero I (1994). Targeted expression of the signaling molecule *decapentaplegic *induces pattern duplications and growth alterations in Drosophila wings. EMBO J.

[B30] Hu Y, Gonzalez-Martinez D, Kim SH, Bouloux PM (2004). Cross-talk of anosmin-1, the protein implicated in X-linked Kallmann's syndrome, with heparan sulphate and urokinase-type plasminogen activator. Biochem J.

[B31] Albuisson J, Pecheux C, Carel JC, Lacombe D, Leheup B, Lapuzina P, Bouchard P, Legius E, Matthijs G, Wasniewska M, Delpech M, Young J, Hardelin JP, Dode C (2005). Kallmann syndrome: 14 novel mutations in KAL1 and FGFR1 (KAL2). Hum Mutat.

[B32] Loidi L, Castro-Feijoo L, Barreiro J, Quinteiro C, Cabanas P, Varela R, Alonso A, Dominguez F, Pombo M (2005). Kallmann's syndrome with a novel missense mutation in the *KAL1 *gene that modifies the major cell adhesion site of the anosmin-1 protein. J Pediatr Endocrinol Metab.

[B33] Rubin GM, Spradling AC (1982). Genetic transformation of *Drosophila *with transposable element vectors. Science.

[B34] Capovilla M, Kambris Z, Botas J (2001). Direct regulation of the muscle-identity gene *apterous *by a Hox protein in the somatic mesoderm. Development.

[B35] Rugarli EI, Ghezzi C, Valsecchi V, Ballabio A (1996). The Kallmann syndrome gene product expressed in COS cells is cleaved on the cell surface to yield a diffusible component. Hum Mol Genet.

[B36] Wieschaus E, Nusslein-Volhard C, Roberts (1986). Looking at embryos in Drosophila, a practical approach.

[B37] Garoia F, Guerra D, Pezzoli MC, Lopez-Varea A, Cavicchi S, Garcia-Bellido A (2000). Cell behaviour of *Drosophila fat *cadherin mutations in wing development. Mech Dev.

